# *N*′-(2,6-Dichloro­benzyl­idene)furan-2-carbohydrazide

**DOI:** 10.1107/S160053681201639X

**Published:** 2012-04-21

**Authors:** Jun Xu

**Affiliations:** a Department of Quality Detection and Management, Zhengzhou College of Animal Husbandry Engineering, Zhengzhou 450011, People’s Republic of China

## Abstract

In the title compound, C_12_H_8_Cl_2_N_2_O_2_, the dihedral angle between the furan and benzene rings is 72.90 (16)°. In the crystal, mol­ecules are linked by N—H⋯O hydrogen bonds, generating *C*(4) chains propagating in [100].

## Related literature

For related structures, see: Okabe *et al.* (1993 ▶); Ohba (1996 ▶); Bakir & Gyles (2003 ▶).
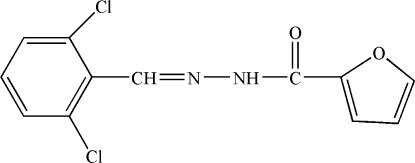


## Experimental

### 

#### Crystal data

C_12_H_8_Cl_2_N_2_O_2_
*M* *_r_* = 283.10
Monoclinic, 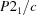

*a* = 4.9046 (3) Å
*b* = 19.1113 (12) Å
*c* = 12.9469 (9) Å
β = 91.565 (5)°
*V* = 1213.10 (14) Å^3^
*Z* = 4
Mo *K*α radiation
μ = 0.53 mm^−1^
*T* = 293 K
0.21 × 0.18 × 0.17 mm

#### Data collection

Bruker SMART CCD diffractometer
Absorption correction: multi-scan (*SADABS*; Bruker, 1998 ▶) *T*_min_ = 0.814, *T*_max_ = 0.847
4654 measured reflections
2464 independent reflections
1679 reflections with *I* > 2σ(*I*)
*R*_int_ = 0.028

#### Refinement

*R*[*F* ^2^ > 2σ(*F* ^2^)] = 0.047
*wR*(*F* ^2^) = 0.126
*S* = 1.01
2464 reflections
163 parameters
H-atom parameters constrained
Δρ_max_ = 0.23 e Å^−3^
Δρ_min_ = −0.30 e Å^−3^

### 

Data collection: *SMART* (Bruker, 1998 ▶); cell refinement: *SAINT* (Bruker, 1998 ▶); data reduction: *SAINT*; program(s) used to solve structure: *SHELXTL* (Sheldrick, 2008 ▶); program(s) used to refine structure: *SHELXTL*; molecular graphics: *SHELXTL*; software used to prepare material for publication: *SHELXTL*.

## Supplementary Material

Crystal structure: contains datablock(s) global, I. DOI: 10.1107/S160053681201639X/hb6741sup1.cif

Structure factors: contains datablock(s) I. DOI: 10.1107/S160053681201639X/hb6741Isup2.hkl

Supplementary material file. DOI: 10.1107/S160053681201639X/hb6741Isup3.cml

Additional supplementary materials:  crystallographic information; 3D view; checkCIF report

## Figures and Tables

**Table 1:** Hydrogen-bond geometry (Å, °)

*D*—H⋯*A*	*D*—H	H⋯*A*	*D*⋯*A*	*D*—H⋯*A*
N1—H1*A*⋯O1^i^	0.86	2.07	2.890 (2)	159

Symmetry code: (i) 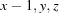.

**Fig. 1.**
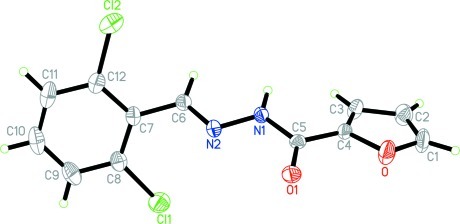
Voew of (I). Displacement ellipsoids are drawn at the 30% probability level. showing the intramolecular hydrogen bonds as dashed lines.

**Fig. 2.**
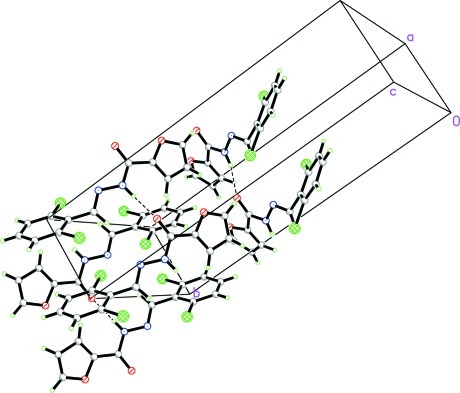
The molecular packing of the title compound,

## supplementary crystallographic information

### Comment

Several phenylhydrazone derivatives have been shown to be potentially DNA-damaging and are mutagenic agents (Okabe *et al.*, 1993). As part of our ongoing studies of these materials, we synthesized the title compound, (I), and the crystal structure is presented herein. In the molecular structure of the compound, the molecular is not planar, the furyl ring makes a dihedral angle of 72.90 (16)° with the benzene ring. Bond lengths and angles are in agreement with other hydrazone derivatives (Ohba, 1996; Bakir & Gyles, 2003).

In the crystal, molecules are linked by N—H···O hydrogen bonds, generating one-dimensional chains.

### Experimental

Furan-2-carbohydrazine (1 mmol, 0.126 g) was dissolved in anhydrous ethanol (10 ml), The mixture was stirred for several minitutes at 351k, 2,6-Dichlorobenzaldehyde (1 mmol, 0.175 g) in ethanol (20 mm l) was added dropwise and the mixture was stirred at refluxing temperature for 2 h. The product was isolated and recrystallized from DMF. Colorless blocks were obtained by slow evaporation of the compound dissoived in a mixture of ethanol and DMF.

### Refinement

All H atoms were positioned geometrically and refined as riding with C—H=0.93 (aromatic), 0.97(methylene) and N—H=0.86 Å, with *U*_iso_(H)=1.2*U*_eq_(CH, CH2 or NH).

### Crystal data

**Table:**

C_12_H_8_Cl_2_N_2_O_2_	*F*(000) = 576
*M**_r_* = 283.10	*D*_x_ = 1.550 Mg m^−^^3^
Monoclinic, *P*2_1_/*c*	Mo *K*α radiation, λ = 0.71073 Å
Hall symbol: -P 2ybc	Cell parameters from 2790 reflections
*a* = 4.9046 (3) Å	θ = 3.2–26.3°
*b* = 19.1113 (12) Å	µ = 0.53 mm^−^^1^
*c* = 12.9469 (9) Å	*T* = 293 K
β = 91.565 (5)°	Block, colorless
*V* = 1213.10 (14) Å^3^	0.21 × 0.18 × 0.17 mm
*Z* = 4	

### Data collection

**Table:**

Bruker SMART CCD diffractometer	2464 independent reflections
Radiation source: fine-focus sealed tube	1679 reflections with *I* > 2σ(*I*)
Graphite monochromator	*R*_int_ = 0.028
ω scans	θ_max_ = 26.4°, θ_min_ = 3.3°
Absorption correction: multi-scan (*SADABS*; Bruker, 1998)	*h* = −5→6
*T*_min_ = 0.814, *T*_max_ = 0.847	*k* = −22→23
4654 measured reflections	*l* = −16→15

### Refinement

**Table:**

Refinement on *F*^2^	Primary atom site location: structure-invariant direct methods
Least-squares matrix: full	Secondary atom site location: difference Fourier map
*R*[*F*^2^ > 2σ(*F*^2^)] = 0.047	Hydrogen site location: inferred from neighbouring sites
*wR*(*F*^2^) = 0.126	H-atom parameters constrained
*S* = 1.01	*w* = 1/[σ^2^(*F*_o_^2^) + (0.0537*P*)^2^ + 0.2769*P*] where *P* = (*F*_o_^2^ + 2*F*_c_^2^)/3
2464 reflections	(Δ/σ)_max_ < 0.001
163 parameters	Δρ_max_ = 0.23 e Å^−^^3^
0 restraints	Δρ_min_ = −0.30 e Å^−^^3^

### Special details

**Table:**

Geometry. All e.s.d.'s (except the e.s.d. in the dihedral angle between two l.s. planes) are estimated using the full covariance matrix. The cell e.s.d.'s are taken into account individually in the estimation of e.s.d.'s in distances, angles and torsion angles; correlations between e.s.d.'s in cell parameters are only used when they are defined by crystal symmetry. An approximate (isotropic) treatment of cell e.s.d.'s is used for estimating e.s.d.'s involving l.s. planes.
Refinement. Refinement of *F*^2^ against ALL reflections. The weighted *R*-factor *wR* and goodness of fit *S* are based on *F*^2^, conventional *R*-factors *R* are based on *F*, with *F* set to zero for negative *F*^2^. The threshold expression of *F*^2^ > σ(*F*^2^) is used only for calculating *R*-factors(gt) *etc*. and is not relevant to the choice of reflections for refinement. *R*-factors based on *F*^2^ are statistically about twice as large as those based on *F*, and *R*- factors based on ALL data will be even larger.

### Fractional atomic coordinates and isotropic or equivalent isotropic displacement parameters (Å^2^)

**Table:**

	*x*	*y*	*z*	*U*_iso_*/*U*_eq_	
Cl1	0.43228 (17)	0.43978 (4)	0.09622 (7)	0.0649 (3)	
Cl2	−0.28672 (16)	0.52357 (5)	0.38143 (7)	0.0679 (3)	
O1	0.5843 (3)	0.68141 (9)	0.10167 (15)	0.0429 (5)	
N1	0.1507 (4)	0.63974 (10)	0.09980 (16)	0.0345 (5)	
H1A	−0.0175	0.6482	0.0838	0.041*	
C5	0.3496 (5)	0.68267 (12)	0.06671 (19)	0.0322 (6)	
N2	0.2201 (4)	0.58266 (10)	0.15913 (16)	0.0342 (5)	
O	0.4344 (4)	0.78012 (11)	−0.04747 (17)	0.0591 (6)	
C7	0.0788 (5)	0.47943 (13)	0.2441 (2)	0.0367 (6)	
C4	0.2594 (5)	0.72916 (12)	−0.0172 (2)	0.0341 (6)	
C12	−0.0570 (5)	0.46354 (15)	0.3342 (2)	0.0452 (7)	
C6	0.0226 (5)	0.54460 (13)	0.1877 (2)	0.0357 (6)	
H6A	−0.1564	0.5581	0.1729	0.043*	
C3	0.0404 (5)	0.72915 (15)	−0.0800 (2)	0.0464 (7)	
H3A	−0.1086	0.6993	−0.0761	0.056*	
C11	−0.0117 (7)	0.40168 (19)	0.3879 (3)	0.0630 (9)	
H11A	−0.1082	0.3920	0.4471	0.076*	
C8	0.2643 (5)	0.42899 (14)	0.2112 (2)	0.0440 (7)	
C9	0.3144 (6)	0.36796 (16)	0.2651 (3)	0.0596 (9)	
H9A	0.4412	0.3358	0.2418	0.071*	
C1	0.3139 (6)	0.81122 (17)	−0.1316 (3)	0.0603 (9)	
H1B	0.3901	0.8477	−0.1686	0.072*	
C10	0.1756 (7)	0.35512 (19)	0.3533 (3)	0.0698 (10)	
H10A	0.2094	0.3141	0.3901	0.084*	
C2	0.0749 (6)	0.78259 (17)	−0.1535 (2)	0.0578 (8)	
H2B	−0.0466	0.7951	−0.2068	0.069*	

### Atomic displacement parameters (Å^2^)

**Table:**

	*U*^11^	*U*^22^	*U*^33^	*U*^12^	*U*^13^	*U*^23^
Cl1	0.0791 (6)	0.0484 (5)	0.0682 (6)	0.0052 (4)	0.0217 (4)	−0.0113 (4)
Cl2	0.0658 (5)	0.0828 (7)	0.0559 (5)	−0.0035 (4)	0.0182 (4)	−0.0036 (5)
O1	0.0292 (9)	0.0457 (11)	0.0537 (12)	−0.0007 (7)	−0.0008 (8)	0.0043 (10)
N1	0.0292 (10)	0.0329 (11)	0.0413 (13)	0.0004 (8)	0.0004 (9)	0.0082 (11)
C5	0.0318 (12)	0.0296 (12)	0.0355 (14)	0.0012 (9)	0.0062 (10)	−0.0048 (12)
N2	0.0384 (11)	0.0289 (10)	0.0352 (12)	−0.0006 (9)	−0.0006 (9)	0.0026 (10)
O	0.0528 (11)	0.0557 (13)	0.0682 (15)	−0.0172 (9)	−0.0062 (10)	0.0223 (12)
C7	0.0410 (13)	0.0336 (13)	0.0352 (14)	−0.0083 (10)	−0.0076 (11)	0.0036 (12)
C4	0.0382 (12)	0.0274 (12)	0.0371 (14)	−0.0015 (10)	0.0076 (11)	0.0011 (12)
C12	0.0477 (15)	0.0479 (17)	0.0399 (16)	−0.0100 (12)	−0.0039 (12)	0.0042 (14)
C6	0.0349 (13)	0.0362 (13)	0.0359 (15)	−0.0011 (10)	−0.0002 (11)	0.0022 (12)
C3	0.0424 (14)	0.0544 (17)	0.0424 (16)	−0.0126 (12)	−0.0027 (12)	0.0150 (15)
C11	0.074 (2)	0.067 (2)	0.0473 (19)	−0.0242 (18)	−0.0090 (16)	0.0222 (18)
C8	0.0497 (15)	0.0348 (14)	0.0470 (17)	−0.0055 (12)	−0.0073 (13)	0.0009 (14)
C9	0.0660 (19)	0.0359 (16)	0.076 (2)	0.0018 (13)	−0.0177 (17)	0.0025 (17)
C1	0.070 (2)	0.0499 (18)	0.061 (2)	−0.0085 (15)	0.0033 (17)	0.0271 (18)
C10	0.085 (2)	0.051 (2)	0.072 (3)	−0.0080 (17)	−0.025 (2)	0.025 (2)
C2	0.0581 (18)	0.064 (2)	0.0505 (19)	0.0017 (15)	−0.0056 (14)	0.0179 (18)

### Geometric parameters (Å, º)

**Table:**

Cl1—C8	1.733 (3)	C12—C11	1.387 (4)
Cl2—C12	1.731 (3)	C6—H6A	0.9300
O1—C5	1.226 (3)	C3—C2	1.410 (4)
N1—C5	1.353 (3)	C3—H3A	0.9300
N1—N2	1.372 (3)	C11—C10	1.363 (5)
N1—H1A	0.8600	C11—H11A	0.9300
C5—C4	1.463 (3)	C8—C9	1.378 (4)
N2—C6	1.275 (3)	C9—C10	1.367 (4)
O—C1	1.362 (4)	C9—H9A	0.9300
O—C4	1.363 (3)	C1—C2	1.318 (4)
C7—C12	1.392 (4)	C1—H1B	0.9300
C7—C8	1.400 (4)	C10—H10A	0.9300
C7—C6	1.466 (3)	C2—H2B	0.9300
C4—C3	1.329 (4)		
			
C5—N1—N2	119.29 (19)	C4—C3—H3A	126.2
C5—N1—H1A	120.4	C2—C3—H3A	126.2
N2—N1—H1A	120.4	C10—C11—C12	119.4 (3)
O1—C5—N1	123.3 (2)	C10—C11—H11A	120.3
O1—C5—C4	123.3 (2)	C12—C11—H11A	120.3
N1—C5—C4	113.4 (2)	C9—C8—C7	122.5 (3)
C6—N2—N1	115.94 (19)	C9—C8—Cl1	117.0 (2)
C1—O—C4	106.2 (2)	C7—C8—Cl1	120.5 (2)
C12—C7—C8	115.8 (2)	C10—C9—C8	119.3 (3)
C12—C7—C6	121.0 (2)	C10—C9—H9A	120.4
C8—C7—C6	123.3 (2)	C8—C9—H9A	120.4
C3—C4—O	109.2 (2)	C2—C1—O	110.8 (3)
C3—C4—C5	132.6 (2)	C2—C1—H1B	124.6
O—C4—C5	117.9 (2)	O—C1—H1B	124.6
C11—C12—C7	122.2 (3)	C11—C10—C9	120.8 (3)
C11—C12—Cl2	119.0 (2)	C11—C10—H10A	119.6
C7—C12—Cl2	118.8 (2)	C9—C10—H10A	119.6
N2—C6—C7	119.7 (2)	C1—C2—C3	106.1 (3)
N2—C6—H6A	120.2	C1—C2—H2B	126.9
C7—C6—H6A	120.2	C3—C2—H2B	126.9
C4—C3—C2	107.7 (2)		

### Hydrogen-bond geometry (Å, º)

**Table:**

*D*—H···*A*	*D*—H	H···*A*	*D*···*A*	*D*—H···*A*
N1—H1*A*···O1^i^	0.86	2.07	2.890 (2)	159

Symmetry code: (i) *x*−1, *y*, *z*.
